# Advancements in genetic circuits as part of intelligent biotherapy for the treatment of bladder cancer: A review

**DOI:** 10.14440/bladder.2024.0044

**Published:** 2025-02-04

**Authors:** Chu Liu, Junlin Lu, Jiajian Lai, Kaiwen Jie, Tianxin Lin, Haifeng Wang, Xu Chen

**Affiliations:** 1 Department of Urology, Sun Yat-sen Memorial Hospital, Sun Yat-sen University, Guangzhou, Guangdong 510120, China; 2 Department of Urology, The Second Affiliated Hospital of Kunming Medical University, Kunming, Yunnan 650101, China

**Keywords:** Bladder cancer, Genetic circuits, Biotherapy, Precise therapy

## Abstract

**Background:**

Bladder cancer poses a significant threat to human health. In recent years, genetic circuit therapy has emerged as a novel alternative for precision tumor treatment, demonstrating promising potential for clinical application. Compared to traditional drugs, genetic circuits – typically carried by plasmids – offer advantages such as modularity, druggability, and shorter drug development cycles. These circuits can identify multiple molecular signals from tumors and integrate them through logic gates to specifically target tumor cells. Furthermore, by assembling effector modules, they can induce specific forms of cell death in tumor cells or alter malignant phenotypes, thereby reshaping the immune microenvironment to produce efficient, durable, and controllable antitumor effects. The urinary system serves as an ideal model for this new therapy due to its accessibility from the outside. Several genetic circuits have already been validated for the treatment of bladder cancer. This review outlined the effectiveness and potential value of genetic circuits in bladder cancer therapy.

**Objective:**

This review focused on the design principles of genetic circuits, their ability to recognize and convert signals, their therapeutic signal output, and the associated delivery vehicles. We also discussed the challenges and future prospects of genetic circuits as a novel form of “intelligent biotherapy.”

**Conclusion:**

The gene circuit can identify multiple signals, processing complex information, and generating multiple effects, thus providing a new approach for personalized treatment of tumors.

## 1. Introduction

Bladder cancer is a lethal disease of the urinary system, with approximately 570,000 new cases and 210,000 deaths reported globally in 2020.^1^ The primary treatment for non-muscle-invasive bladder cancer (NMIBC) is transurethral resection of bladder tumor (TURBT), followed by intravesical instillation. Despite the introduction of non-invasive urine diagnostic tools, such as UriFind and Bladder Epicheck for early diagnosis and recurrence prediction for NMIBC patients,^2^-^5^ the 5-year recurrence rate after TURBT remains significant, ranging from 31% to 78%, with disease progression occurring in 10 – 30% of patients,^6^,^7^ Radical cystectomy is the main surgical treatment for muscle-invasive bladder cancer, but it significantly affects patients’ quality of life. Conventional chemotherapies, such as cisplatin, generally result in a high incidence of adverse effects due to their poor discrimination between normal and tumor cells. These drugs, which target a single pathway or mechanism, can also facilitate the development of drug resistance.^8^ Immune checkpoint inhibitors (ICIs) and antibody-drug conjugates (ADCs) represent promising pathways to the targeted treatment of bladder cancer.^9^,^10^ However, ICIs are ineffective in treating tumors with minimal immune cell infiltration,^11^ and ADCs are applicable only to a small number of patients due to their dependence on the presence of specific targets.^12^ Furthermore, patients’ responses to drugs vary considerably, underscoring the limitations of traditional chemotherapeutic agents. Moreover, the development of conventional pharmaceuticals necessitates exhaustive pharmacological screening and prolonged development periods, which further complicate drug development and application. Given these challenges, tumor therapy demands a shift toward more personalized and tailored strategies. By designing precise therapeutic strategies based on the specific characteristics of individual tumors, drug efficacy can be enhanced, adverse effects diminished, and drug resistance mitigated.

**Figure 1:**
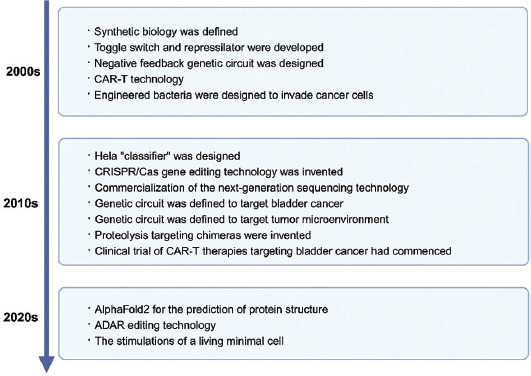
Milestones in the development of synthetic biology in tumor therapy Abbreviations: ADAR: Adenosine deaminases acting on ribonucleic acids; CAR-T: Chimeric antigen receptor T-cells; Cas: CRISPR-associated protein; CRISPR: Clustered regularly interspaced short palindromic repeats.

The advent of “toggle switches” and “suppressors” at the beginning of the 21^st^ century marked the formal establishment of synthetic biology as a distinct field of study.^13^,^14^ Significant advancements have been made in tumor therapy through the application of synthetic biology, including genetic circuits, immune cell modification, and bacterial engineering (Figure 1). In recent years, genetic circuit therapy has emerged as a novel technology in precision medicine, exhibiting considerable potential in the treatment of bladder cancer. Compared to traditional therapies, genetic circuits offer several advantages: (i) Modularity: The modules within genetic circuits can be designed independently after decoupling, allowing for easy substitutions to perform various functions;^15^ (ii) druggability: genetic circuits can specifically respond to the expression levels of nucleic acids and proteins, unaffected by small-molecule binding pockets, enabling the targeting of traditionally “undruggable” targets; (iii) short development cycle: with established genetic circuit frameworks, the development of new targets requires only the testing and optimization of individual modules, leading to rapid research and development. These features allow for the entire process – tumor feature identification, module screening, and genetic circuit assembly – to be completed in a short time frame for individual patients, providing a new approach for achieving precision tumor treatment. This review discussed recent advances in genetic circuit therapy for bladder cancer.

## 2. Search strategy

We conducted a comprehensive search across academic databases, including PubMed and Web of Science, to identify advances in genetic circuit construction and delivery technologies in tumor therapy. The search was performed using a combination of keywords and Boolean operators, in terms such as “bladder cancer,” “genetic circuits,” “synthetic biology,” and “delivery technology.” We excluded brief correspondence, case reports, editorials, reviews, letters to the editor, research protocols, and papers not published in English.

Researchers independently extracted data on genetic circuit design, delivery vectors, and application effectiveness from the selected articles to assess their therapeutic potential for bladder cancer. Any discrepancies in data extraction were resolved through consensus among the co-authors.

**Figure 2:**
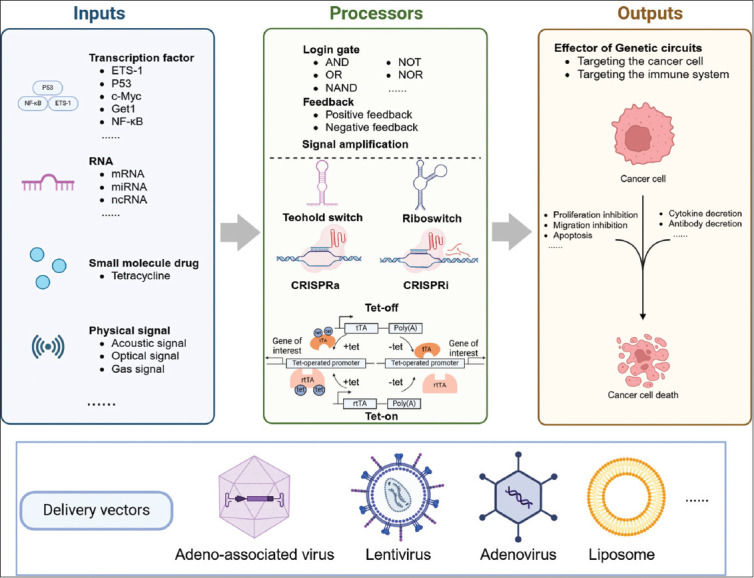
Basic principles of genetic circuit design Abbreviations: CRISPRa: CRISPR activation; CRISPRi: CRISPR inactivation; ETS-1: V-ets avian erythroblastosis virus E26 oncogene homolog 1; Get1: Grainyhead-like epithelial transactivator 1; mRNA: Messenger RNA; miRNA: MicroRNA; NAND: NOT-AN; ncRNA: Non-coding RNA; NF-κB: Nuclear factor kappa-light-chain-enhancer of activated B cells; Poly(A): Poly-adenine tail; P53: Tumor protein P53; RNA: Ribonucleic acid; rtTA: Reverse tetracycline transactivator; tet: Tetracycline; tTA: Tetracycline transactivator.

Genetic circuits are a pivotal aspect of synthetic biology. These circuits consist of various genetic devices comprising diverse regulatory elements and the genes they regulate.^16^ The modules within genetic circuits fall into three primary categories: signal detection, signal processing, and signal output (Figure 2). The tumor signal detection module, or sensor, is responsible for identifying aberrant tumor targets, including nucleic acids, proteins, and signals in the tumor microenvironment. The signal processing module, or processor, integrates and processes multiple input signals. Finally, the signal output module, or effector, regulates intracellular signaling networks or the tumor microenvironment to achieve therapeutic goals.

## 3. Sensors of genetic circuits

The recognition of transcription factors is a classic form of genetic circuit sensors, enabling the distinction between anticancer or pro-cancer transcription factor signals. The binding of transcription factors to deoxyribonucleic acid (DNA) is sequence-specific and can be employed in the construction of artificial synthetic promoters. Zhan *et al*.^17^ synthesized P53-binding enhancer regions (P53BERs), which can specifically recognize and bind the transcription factor P53. These enhancer regions were positioned upstream of the core promoter simian virus 40 (SV40), thereby forming a P53 sensor. In bladder cancer cells, the P53 sensor detects signals associated with P53 deficiency and subsequently triggers the expression of diphtheria toxin through clustered regularly interspaced short palindromic repeats (CRISPR)/CRISPR-associated protein (Cas) 9. The construction of artificial promoters can also be employed to identify discrepancies in the expression of transcription factors. Transcription factor v-ets avian erythroblastosis virus E26 oncogene homolog 1 (ETS-1), a transcription factor that is highly expressed in bladder cancer cells, was targeted through synthetic biology methods involving the “simplify-test-concatenate” approach. This event resulted in the construction of a bladder cancer-specific artificial promoter aligned with the expression level of the the transcription factor ETS-1, allowing for the precise identification of bladder cancer cells and promoting their apoptosis.^18^ Several synthetic promoters have been designed to target bladder cancer, including the human telomerase reverse transcriptase (hTERT) promoter and the bladder-specific promoter of the human uroplakin II gene (hUPII). These promoters have demonstrated promising results in recognizing abnormal signals in bladder cancer.^19^ CRISPReader represents a novel, non-promoter-dependent gene expression regulation technology developed based on CRISPR technology. It specifically responds to the presence of bladder cancer-specific transcription factors by selectively deleting promoters other than the bladder tissue-specific promoter UPII and the cancer-specific promoter TERT, while retaining the associated TATA box. Once the TATA box initiates transcription, the single guide ribonucleic acid (sgRNA) produced during this process can bind endonuclease-deficient Cas9 (dCas9)-based transcriptional activators to the upstream sequences of the TATA box. This amplification allows CRISPReader technology to amplify the identification of bladder cancer tumor signals, thereby constructing a genetic circuit capable of accurately identifying and killing bladder cancer cells.^20^,^21^ Genetic circuits can also recognize progressive tumor cells through specific sensors, such as ETS translocation variant 4,^22^ heat shock factor protein 1,^23^ and GATA-binding factor 6,^24^ which have been identified as lymphatic metastasis-associated genes. Conventionally, transcription factors lack small-molecule binding pockets and are considered “undruggable” targets. Genetic circuits provide an effective solution for targeting functionally abnormal transcription factors.

MicroRNAs (miRNAs) are another class of carcinogenic molecules, characterized as non-coding ribonucleic acids (RNAs), measuring approximately 22 nucleotides in length. Mechanistically, miRNAs form base-pairing interactions with messenger RNAs (mRNAs), leading to the degradation of the targeted mRNAs.^25^ Based on this principle, miRNA binding sites with high degradation efficiency can be artificially designed to respond to varying miRNA levels. The sensitivity of the response can be adjusted by altering the number of tandem binding sites. Xie *et al*.^26^ devised an RNA interference (RNAi)-based logic circuit for identifying specific tumor cells. Initially, a set of Henrietta Lacks (HeLa)-high and HeLa-low miRNA markers was identified, and a sensor was designed that contains a “double inversion” module, which activates the output exclusively in the presence of HeLa-high miRNAs. A comprehensive genetic circuit was subsequently constructed, comprising a series of sensors for both high- and low-expression markers associated with HeLa cells. The output is activated when all high-expression markers in HeLa cells are simultaneously present, triggering apoptosis. If the expression level of any marker is low, the output is inhibited, thus preventing injury to healthy cells. This “HeLa cell classifier” can effectively distinguish HeLa cells from other cells and induce apoptosis by sensing the expression of five miRNAs. In the synthetic RNA-based miRNA-responsive CRISPR-Cas9 system, the mechanism by which miRNAs recognize mRNAs is also crucial. The system achieves precise regulation of Cas9 genome editing activity by designing complementary sequences of specific miRNAs in the 5’ untranslated region of the mRNA encoding Cas9. This recognition mechanism is able to detect and respond to discrepancies in the activity of endogenous miRNAs in heterogeneous cell populations, allowing for varying degrees of control over genome editing.^27^ Cell classifiers based on miRNA-mRNA interactions can integrate multiple miRNA signals and are small in size, facilitating the construction of complex genetic circuits. However, several challenges remain with miRNA-based genetic circuits, including the potential for the miRNA module to respond to imperfectly matched miRNAs, which can result in output leakage.

Chimeric antigen receptor T (CAR-T) cell therapy is a revolutionary cancer treatment method that involves genetically engineering T cells to recognize and attack cancer cells. This approach holds particular promise for the treatment of bladder cancer. The modular synthetic Notch (synNotch) receptor, comprising the Notch extracellular domain, transmembrane domain, and Notch intracellular structural domain, has a robust capacity for specific tumor antigen recognition.^28^ CAR-T cells equipped with this receptor have shown significant advantages in overcoming the challenges of tumor heterogeneity and enhancing the efficacy of solid tumor treatments.^29^ At present, several clinical trials on CAR-T cell therapy for bladder cancer are underway, including a Phase I study (NCT03740256) that explores the combination of oncolytic adenovirus therapy with CAR-T cells to treat human epidermal growth factor receptor 2-positive bladder cancer. Although the results of these trials have not yet been reported, they provide important insights for future treatment strategies.

## 4. Processors in genetic circuits

The integration of processing functions into genetic circuits enables the analysis and manipulation of tumor signals. Common design concepts employed in genetic circuits include logic gates and signal amplification. In addition, various components are frequently utilized as processors, including toehold switches, riboswitches, adenosine deaminase acting on ribonucleic acid (ADAR) switches, CRISPR-Cas systems, CRISPR/dCas systems, small-molecule chemoswitches, and light-induced switches, all of which are discussed in this section.

### 4.1. Logic gates and signal amplification

Tumor formation relies on the presence of multiple oncogenic signals. Genetic circuits that integrate these signals provide a precise method for distinguishing tumor cells and identifying their unique cellular functions. The fundamental approach to signal integration entails the logical operations of “AND,” “OR,” and “NOT” gates. The “AND” gate is the most commonly used logic gate, activating outputs exclusively when multiple tumor signals are present simultaneously. The CRISPR/Cas system is frequently employed in constructing logic gates. Cas9 and sgRNA, expressed via artificial bladder cancer-specific hUPII and hTERT promoters, respectively, serve as “AND” gates to promote the recognition of bladder cancer specificity.^30^ The implementation of “NOT” gates, constructed by the loss of tumor-suppressor transcription factors in tumor cells, represents a pivotal strategy for avoiding leaky effector expression. Oncogenic transcription factors can stimulate the expression of CRISPR activation (CRISPR/dCas9-based endogenous gene transcription effector), while transcription factors responsive to P53 signaling to induce the expression of the anti-CRISPR protein, enabling targeted initiation of genetic circuit expression in P53-deficient tumor cells.^31^ Furthermore, the utilization of the split Cas12a system to engineer genetic circuits facilitates the recognition of three to four signals in tumor cells, including combinatorial responses to microenvironmental signals and exogenous small-molecule signals.^32^

The construction of multilayered computational gene networks offers a more intricate and adaptable approach to gene regulation than traditional “logic gates.” Shao *et al*.^33^ developed tristate buffers that include “downstream switches” for direct translational control and “upstream switches” that regulate transcription. They used the “Grazoprevir switch” to modulate translation between the “ON” state (BUF) and the “OFF” state (NOT),^34^ and the “vanillic acid switch” to control transcription in the “ON” state (IF1) or “OFF” state (IF0). This dual-switch system enables the implementation of four Boolean logic operations: BUFIF1, NOTIF1, BUFIF0, and NOTIF0. Moreover, the complexity of Boolean logic operations within mammalian cells can be significantly expanded by incorporating additional tristate buffers, thereby enabling the execution of over ten distinct complex Boolean functions. The tristate-based logic synthesis for genetic circuits proposed in this study provides technological innovations in the field of cellular computing.

The activation efficacy of synthetic promoters is typically modest, exhibiting several- to a dozen-fold greater capacity to distinguish between normal and tumor cells. Signal amplification mechanisms, including positive feedback, multistep signal transduction, and multiple outputs, are employed to amplify weak signals in genetic circuits. This amplification ensures the generation of sufficient output signals to eliminate tumor cells. Zhan *et al.*,^20^ Liu *et al*.^21^ constructed signal amplifiers by ligating Cas9 to the transcriptionally activated structural domain VP64 to form fusion proteins. They further enhanced the translation of mRNAs via RNA activators, amplifying the expression of downstream genes by approximately two-fold compared to conventional genetic circuits. Dong *et al*.^35^ designed a self-amplified dual-input synthetic genetic circuit to drive the expression of fusion proteins herpes simplex virus-encoded protein VP16-Docs and transcription factor GAL4-Coh2 through two tumor-specific promoters. Due to the high affinity of Docs and Coh, the two fusion proteins can form a transcriptional complex with RNA polymerase, which can bind to five Upstream Activating Sequence (UAS) domains combined with the late adenoviral promoter to produce GAL4-VP16 and therapeutic signals, respectively. Furthermore, GAL4-VP16 can reactivate itself and the therapeutic signal output element, thereby achieving positive feedback signal amplification. This approach increases the sensitivity of tumor recognition by approximately ten-fold while maintaining high specificity at a level of 16. In addition, an incoherent feedforward circuit can be constructed based on miRNAs. In response to the input signal, miRNAs and target mRNAs containing miRNA binding sites are co-expressed. By regulating the binding strength of miRNAs and their binding sites, the strength of the output signal can be controlled. In conclusion, the integration and processing of multiple signals ensure the efficacy and specificity of genetic circuits, which are crucial elements in genetic circuit design.

### 4.2. Toehold switch

A toehold switch is a genetic element composed of nucleic acids based on the Watson-Crick base pairing principle. Theoretically, it can recognize any sequence of RNA, including mRNAs, miRNAs, and non-coding RNAs. The fundamental structure of a toehold switch comprises three distinct strands: a substrate strand, a substitution strand, and an invasion strand. When the concentration of the invasion strand (containing the target sequence) is relatively low, the substrate strand binds with the substitution strand, effectively closing the toehold switch. Conversely, as the concentration of the invasion strand increases, the toehold region within its sequence begins to bind to the complementary toehold region in the substrate strand. This gradual replacement of the substitution strand ultimately activates the toehold switch. The toehold switch can accurately sense the concentration of target RNA. By modifying the binding sequence of the substrate strand and the invasion strand, the system can detect different RNA sequences, which has been successfully applied to living-cell imaging.^36^ Furthermore, the toehold switch can be adapted based on the sgRNA, with the toehold-sgRNA serving as the input signal and the CRISPR/Cas system functioning as the processor and output, thus enabling the modification of genetic circuits. This allows for greater programmability of genetic circuits.^37^ The system can be tailored to respond to varying RNA levels within the cell by designing specific substitution strand sequences.

### 4.3. Riboswitch

A riboswitch is composed of two distinct components: an aptamer and an expression platform. The aptamer functions by forming a high-affinity bond with a specific ligand, such as a single-stranded nucleic acid molecule. This interaction induces a conformational change in the expression platform.^38^ Gene expression is regulated through conformational changes that occur after the aptamer recognizes the corresponding ligand, involving the expression platforms. In the field of genetic circuit design, a typical example is the integration of a riboswitch into a sgRNA. Zheng *et al*.^39^ integrated nuclear factor kappa-light-chain-enhancer of activated B cells (NF-κB) aptamers with sgRNAs that target opa-interacting protein 5 (OIP5). In the absence of NF-κB signaling, the sgRNAs pair with antisense stems, resulting in the loss of their ability to bind the target DNA. On recognition of NF-κB, the guide region of the sgRNA is exposed, enabling it to bind and inhibit the target gene OIP5, thereby reversing the resistance of bladder cancer cells to vincristine. High-throughput screening of specific aptamers for the treatment of bladder cancer can further improve the targeting capacity of genetic circuit elements. Yan *et al*.^40^ employed Systematic Evolution of Ligands by Exponential Enrichment to identify aptamers that can be specifically recognized and internalized by bladder cancer cells. The aptamer was then employed in the development of nucleic acid aptamer-chemotherapy combination with targeted drugs carrying epirubicin. It is anticipated that the future integration of multiple aptamers through genetic circuits will facilitate the digital processing of multiple aptamer signals, thereby enhancing the precision and safety of treatment.

### 4.4. Adenosine deaminases acting on ribonucleic acids

Adenosine deaminases acting on ribonucleic acid are a family of RNA-binding proteins that convert adenine (A) to hypoxanthine (I), which in turn affects processes such as RNA splicing, stability, localization, and translation.^41^-^43^ In eukaryotic cells, hypoxanthine (I) is typically recognized as guanine (G) during translation. Recent studies have leveraged this principle to alter the termination codon UAG to the codons UIG or UGG for tryptophan, thereby enabling the expression of downstream proteins. Qian *et al*.^44^ were the first to develop CellREADR, an ADAR-based RNA recognition tool that responds to intracellular mRNAs in the form of linear RNA. On expression, the termination codon UAG is edited by ADAR, resulting in the expression of a downstream fluorescent signal. Moreover, the activation efficiency of this receptor can be enhanced by up to 277-fold through the optimization of the 5’ end protein region length, the optimization of the nucleic acid length of the receptor region, and the addition of the MS2 stem-loop.^45^ The ADAR-based recognition of *in vivo* RNA does not depend on the secondary structure of the RNA to perform its function. Moreover, it exclusively produces editing at the RNA level, which is considered safer. However, its activation efficiency is influenced by the expression of ADAR in target cells.

### 4.5. Clustered regularly interspaced short palindromic repeats/endonuclease-deficient CRISPR-associated protein system

The CRISPR/dCas system is a gene-regulating tool based on CRISPR technology. It utilizes inactivated Cas proteins that specifically bind to, but do not cleave, the target DNA or RNA sequences.^46^ In a study by Cao *et al.*,^47^ inhibition of bladder cancer cells was achieved by fusing dCasX to the transcriptional repressor structural domain Krüppel associated box (KRAB) to silence the oncogene c-Myc, or by fusing dCasX to the potent transcriptional activator domain viral protein R to upregulate the tumor suppressor gene TP53. Li *et al*.^48^ reported that the combined inhibition of both the transcriptional coactivators CREB-binding protein (CBP) and histone acetyltransferase p300 induced apoptosis in bladder cancer cells. To achieve this, they utilized the dCas9-KRAB system to simultaneously downregulate CBP and p300 expression, thereby inducing apoptosis of bladder cancer cells *in vitro*. Furthermore, the CRISPR/dCas system can be employed to modify oncogenes at the epigenetic level, thereby suppressing the malignant phenotype of bladder cancer cells. c-MYC mRNA methylation has been linked to poor prognosis in bladder cancer patients. To target c-MYC mRNA, Su *et al*.^49^ fused the demethylation enzyme adiponectin and the obesity-associated protein to the C-terminal end of the catalytically inactive dCas13b protein. This approach effectively elicited c-MYC mRNA demethylation and inhibited c-MYC transcription and expression.

### 4.6. Chemical and physical switches

The switches mentioned above serve as “intelligent sensors” for specific markers both inside and outside the cell. The introduction of chemical or physical switches allows the cell to respond to external signals, thereby regulating the “ON” or “OFF” state of genetic circuits.

The tetracycline (Tet)inducible element is a commonly used small-molecule chemical switch, which can be categorized into two operational modes: the Tet-on inducible gene system and the Tet-off inducible gene system. The Tet-on inducible gene system has been widely validated for its applications in bladder cancer treatment.^50^-^54^ Chen *et al*.^53^ employed the Tet-on inducible gene system to regulate the expression of short hairpin RNAs targeting the oncogene hypoxia-inducible factor-1α antisense ribonucleic acid 2, facilitating the dose-dependent inhibition of bladder cancer cell progression. Peng *et al*.^54^ utilized the Tet-on inducible system to regulate Cas9 expression in CRISPR/Cas9 system, enabling the targeting of bladder cancer-associated long non-coding RNAs and subsequent inhibition of malignant biological behaviors in bladder cancer. In recent years, several small-molecule chemical switches, such as protocatechuic acid.^55^ and sodium ferulate,^56^ which are more suitable for use in humans, have been developed and validated in mammalian models. These chemical switches are expected to promote the clinical application of genetic circuits.

In addition to chemical genetic switches, physical switches can be constructed to sense physical signals. Optogenetic switches rely on light-induced deformation,^57^ cleavage,^58^ oligomerization, or depolymerization^59^ of photosensitive proteins, which can regulate the opening or closing of genetic circuits in response to changes in light signals. The Avena sativa phototropin 1 (AsLOV2) protein,^60^ which exposes its Jα helical structure on blue light stimulation, cryptochrome 2–cryptochrome-interacting basic-helix-loop-helix 1,^61^ which undergoes dimerization, negative magnet (nMag)-positive magnet (pMag),^60^ and Vivid^62^ have all been validated for their applications in the treatment of urological tumors. These optogenetic switches offer several advantages, including low background expression, minimal off-target effects, and enhanced safety and controllability, which collectively facilitate precise gene editing in bladder cancer cells. Moreover, additional physical signals, including acoustic and aerogenetic switches, can be employed to modulate the opening and closing of genetic circuits.^63^,^64^ These artificial switches provide control over genetic circuits, allowing for the regulation of treatment intensity and duration. They also lay the foundation for the construction of more precise, safe, and effective gene circuits for urological tumors.

## 5. Effectors of genetic circuits

The efficacy of traditional antitumor drugs is limited by their ability to target only a single tumor characteristic, which can result in tumor resistance. Furthermore, the use of multidrug combinations has been shown to increase the incidence of adverse reactions. The modular design of genetic circuits permits the replacement of effector elements according to different tumor features or the simultaneous expression of multiple effectors, thereby achieving composite functions. Apoptosis can be induced by specifically expressing the apoptosis gene BCL2-associated X (Bax) in tumor cells or by inhibiting the expression of B-cell CLL/lymphoma 2 (BCL2) and survivin.^18^,^30^,^52^,^60^ Alternatively, proliferation can be inhibited through the expression of the cell cycle inhibitory protein p21 and the tumor suppressor p53.^21^,^30^,^61^ In addition, the expression of E-cadherin or β-catenin, among other factors, can inhibit tumor cell migration.^30^,^50^,^65^,^66^ Moreover, the effect of chemotherapy can be enhanced by downregulating the drug resistance gene OIP5.^39^ Genetic circuit effectors can also modulate therapeutic effects in target cells at the posttranslational level. A recent study developed synthetic protein circuits for the programmable control of mammalian cell apoptosis or pyroptosis through protein hydrolysis, providing a novel approach for selectively eliminating tumor cells.^67^ In conclusion, these effectors can be adapted to the specific characteristics of the patient’s tumor, enabling the targeting of highly metastatic and drug-resistant tumor cells.

In recent years, therapeutic modalities targeting the immune system have demonstrated long-lasting antitumor efficacy. Similarly, genetic circuits can be engineered to activate the antitumor functions of immune cells. The application of genetic circuits to modify T cells into cytokine “production factories” *in vivo* can substantially increase the concentrations of cytokines at the tumor site.^68^ The assembly of interleukin (IL)-2, for instance, can markedly elevate local IL-2 levels in tumors, stimulating antitumor T-cell immunity and enhancing the functionality of CAR-T cells.^68^ The combination of cytokines and antibodies, including IL-12, chemokine (C-C motif) ligand (CCL) 21, and the ICI anti-programmed cell death protein 1, can be used to remodel the immune microenvironment in a multifaceted manner, addressing immune cell chemotaxis, activation, and depletion.^69^ In line with CAR-T cell design principles, chimeric antigen receptors can be reverse-engineered on tumor cells to target and activate immune cells, thereby achieving specific recognition and destruction of tumor cells.^69^ The local release profile of genetic circuits is the bedrock of therapeutic signal functionality. Consequently, the augmentation of cytotoxic or immune-activating factors can be employed to modulate local therapeutic effects and minimize systemic side effects through this technology.

## 6. *In vivo* therapeutic delivery of genetic circuits

A key challenge in transitioning genetic circuit therapy from the laboratory to clinical practice is the development of effective methods for delivering genetic circuits into the body. An ideal delivery vector should preserve the structural integrity of genetic circuits before cellular internalization and facilitate their release across the target cell membrane.^70^ The current vectors utilized for genetic circuit delivery are primarily classified into two categories: viral vectors and non-viral vectors.^70^ Viral vectors include adeno-associated viruses (AAVs), adenoviruses, lentiviruses, and lysoviruses. Lentiviral vectors, primarily derived from the human immunodeficiency virus, present an increased risk for *in vivo* application and are predominantly utilized in *in vitro* studies. Recombinant adenovirus has been employed to treat Bacillus Calmette–Guerin non-responsive NMIBC patients (NCT02773849). However, adenovirus is highly immunogenic and can activate the immune system, which may result in adverse reactions, including local tissue inflammation or systemic immune responses.^71^ In contrast, AAVs are less immunogenic, more efficient in delivery, and suitable for long-term genetic circuit expression *in vivo*. They have been clinically used for treating ophthalmic and muscle diseases.^72^,^73^ However, the AAV has a capacity of only 4.7 kb, which restricts its ability to deliver complex genetic circuits. Consequently, complex circuits require the codelivery of multiple AAVs or the use of simplified genetics.^21^ Lysogenic viruses are capable of selective replication within tumor cells and can lyse these cells, releasing viruses that subsequently invade and infect other tumor cells, thereby facilitating the delivery of genetic circuits between tumor cells.^74^

Non-viral vehicles encompass a range of materials, including cationic polymers, nanoparticles, and liposomes. These vehicles offer significant advantages in terms of safety, designability, and capacity, rendering them ideal for genetic circuit delivery. Compared to viral vectors, non-viral vehicles are more abundant, less immunogenic, and do not carry the risk of infection or gene integration that viral vectors present. Fan *et al*.^75^ designed liposomes as delivery vehicles for CRISPR/Cas12a genetic circuits targeting BCL2, vascular endothelial growth factor receptor 2, and survivin, and they were delivered in high-capacity, multilayered systems and significantly inhibited the malignant phenotype of bladder cancer cells. Similarly, Zheng *et al*.^39^ developed cationized human serum albumin nanoparticles to co-deliver genetic circuits and vincristine, resulting in enhanced cytotoxicity against bladder cancer, including vincristine-resistant bladder cancer cell lines, in human tumor organoid models and in murine models that replicate the *in vivo* environment. Despite these advantages, non-viral vectors typically exhibit lower gene delivery efficacy *in vivo* compared to viral vectors. This limitation is attributed to the absence of the evolutionary advantage associated with the long-term host adaptation observed in biogenic viruses. A recent study developed siloxane-based ionizable lipids and formulated siloxane-incorporated lipid nanoparticles for tissue-specific mRNA delivery to the lungs, spleen, and liver.^76^ However, developing vectors for bladder-specific targeting remains a significant challenge in genetic circuit research. Future studies should focus on devising innovative strategies to improve the safety and efficacy of genetic circuit delivery systems.

## 7. Genetic circuit assembly

The construction of a complete genetic circuit, assembled from the aforementioned components, facilitates the precision treatment of urological tumors. On transfection of the CRISPR/Cas9-based “AND” gate genetic circuit into bladder cancer cells via plasmids, the hUPII promoter, and hTERT promoter are employed to drive the expression of Cas9 and sgRNA, respectively. These events effectively inhibit bladder cancer cell proliferation and migration while promoting apoptosis through the production of p21, Bax, and E-cadherin.^30^ Optogenetic technology, with its remarkable advantages of non-invasiveness, high reversibility, and precise spatiotemporal control, has shown great promise in the treatment of bladder cancer. The light-controlled activation of Cas9 enzyme activity was achieved by splitting the survivin-targeting Cas9 protein into two independent modules, which were then fused with the nMag and pMag proteins, which could form heterodimers under blue light irradiation. Moreover, the unique properties of the AsLov2 system were combined with an innovative design to fuse the Von Hippel–Lindau (VHLL) ligand – a key ligand for E3 ligases – to the C-terminal end of the Jα helix. This design not only allows for precise control of the Cas9 protein, enabling it to be complexed and activated under blue light but also promotes the downregulation of survivin gene expression. In addition, the VHLL ligand can be intelligently exposed or hidden by modulating changes in the structure of AsLov2. Under blue light exposure, the Cas9 protein efficiently assembles and acts on the survivin gene, reducing its transcription. The conformational change in AsLov2 enables the VHLL ligand to be fully exposed and efficiently recruited to the E3 ligase, greatly enhancing the degradation of the survivin protein bound to AsLov2 N-terminal nanobodies through the ubiquitin–proteasome pathway. This process leads to the effective induction of apoptosis and significant inhibition of the proliferation and migration of bladder cancer cells. Table 1 illustrates the typical studies of genetic circuits utilized in the treatment of bladder cancer.

**Table 1:** Summary of major studies of therapeutic genetic circuits for bladder cancer

Study	Input signal	Signal processing	Therapeutic signal	Delivery vehicle
Zhan *et al*.^17^	P53	CRISPR/Cas9	Diphtheria toxin	Lipofectamin*^a^*
Liu *et al*.^18^	ETS-1	Artificial hTERT promoter	Bax↑, BCL2↓	Lipofectamin*^a^*
Liu *et al*.^21^	c-Myc, Get1	CRISPReader	p21↑, Bax↑, E-cadherin↑	Lipofectamine, AAV*^b^*
Liu *et al*.^30^	hTERT, hUPII	CRISPR/Cas9	p21↑, Bax↑, E-cadherin↑	Lipofectamine*^a^*
Zheng *et al*.^39^	NF-κB	CRISPR/Cas9	NF-κB↓, OIP5↓, Bax↑, BAK1↑, BIK↑, cytochrome P450↓, UGT↓	Lipofectamine, LV*^b^*
Cao *et al*.^47^	Not available	CRISPR/dCasX-KRAB or CRISPR/dCasX -VPR	c-MYC↓↓, TP53↑	Not mentione*^d^*
Li *et al*.^48^	hTERT, hUPII	CRISPR/dCas9-KRAB	CBP↓, p300↓	Lipofectamine*^a^*
Su *et al*.^49^	Not available	CRISPR/dCas13b-FTO	MYC	LV*^b^*
Zhan *et al*.^50^	Tetracycline	Tet-on	β-catenin↓, HIF-1α↓	Lipofectamine*^a^*
Li *et al*.^51^	Tetracycline	Tet-on	lncRNA CCAT2↓	Lipofectamine*^a^*
Lin *et al*.^52^	Tetracycline	Tet-on	hTERT↓, BCL2↓	Lipofectamine*^a^*
Chen *et al*.^53^	Tetracycline	Tet-on	HIF1A-AS2↓	LV*^a^*
Peng *et al*.^54^	Tetracycline	Tetracycline-inducible CRISPR/Cas9 system	PVT1↓, ANRIL↓	Lipofectamine*^a^*
Deng *et al*.^60^	Blue light	AsLOV2, nMag-pMag	Survivin↓	LV*^b^*
Lin *et al*.^61^	Blue light	CRY2-CIB1	P53↑	Lipofectamine*^a^*
Qi *et al*.^62^	Blue light	Vivid	lncRNA MALAT1↓	Lipofectamine*^a^*
Zhuang *et al*. 2021^77^	hTERT	CRISPR/Cas13d	MYC↓	Lipofectamine*^a^*
Liu *et al*., 2018^78^	NF-κB	Aptamer	Bax↑, p21↑, Bcl2↓, c-myc↓	Lipofectamine*^b^*

Notes:

a stands for *in vitro* study,

b stands for both *in vitro* and *in vivo* studies. Abbreviations: AAV: Adeno-associated virus; ANRIL: antisense non-coding RNA in the INK4 locus; AsLOV2: Avena sativa light, oxygen, or voltage 2; BAK1: BCL2 antagonist/killer 1; Bax: BCL2-associated X, apoptosis regulator; BCL2: B-cell CLL/lymphoma 2; BIK: BCL2-interacting killer; Cas: CRISPR-associated protein; CBP: CREB-binding protein; CRISPR: Clustered regularly interspaced short palindromic repeats; CCAT2: Colon Cancer Associated Transcript 2; CIB1: Cryptochrome-interacting basic-helix-loop-helix 1; CRY2: Cryptochrome 2; dCasX: CRISPR/nuclease-deficient; dCas9: Endonuclease-deficient Cas9; dCas13b: Catalytically inactive Cas13b; ETS-1: V-ets avian erythroblastosis virus E26 oncogene homolog 1; FTO: Fat mass and obesity-associated protein; Get1: Get1: Grainyhead-like epithelial transactivator 1; HIF-1α: Hypoxia-inducible factor-1α; HIF1A-AS2: HIF1A antisense ribonucleic acid 2; hTERT: Human telomerase reverse transcriptase; hUPII: Human uroplakin II; KRAB: Krüppel associated box; lncRNA: Long non-coding RNA; LV: Lentivirus; MALAT1: Metastasis associated lung adenocarcinoma transcript 1; NF-κB: Nuclear factor kappa-light-chain-enhancer of activated B cells; nMag: Negative magnet; OIP5: Opa interacting protein 5; PVT1: Plasmacytoma variant translocation 1; pMag: Positive magnet; p21: Cyclin-dependent kinase inhibitor 1; p300: Histone acetyltransferase p300; P53: Tumor protein P53; TP53: Gene that encodes for the p53 tumor suppressor protein; UGT: Uridine diphosphate-glucuronosyltransferase; VPR: Viral protein R.

## 8. Summary and prospects

Genetic circuits provide a novel approach to individualized treatment for bladder cancer patients. These circuits can recognize multiple signals within and outside bladder cancer cells, make logical decisions, and directly regulate the biological behaviors of tumor cells, such as proliferation, migration, or apoptosis. In addition, they can modulate the tumor microenvironment to enhance antitumor immunity, thereby achieving precision tumor treatment. Genetic circuits enable therapeutic drugs to perform complex calculations and engage in decision-making, mimicking those in the brain, and are thus referred to as “intelligent biological therapy” in the field of precision tumor treatment.

However, genetic circuits still face several challenges. The assessment of their efficacy is primarily limited to *in vitro* studies and animal models, with a pressing need for more evidence to ascertain their ability to differentiate between tumor cells and normal cells, as well as to produce therapeutic effects in humans. The use of viral vectors for genetic circuit delivery has demonstrated safety and efficacy in clinical trials involving intravesical viral therapy. Nevertheless, patients with significant tumor burden and metastasis require systemic treatment. The safety of *in vivo* applications of CRISPR systems and viral vectors necessitates comprehensive evaluation. Therefore, there is an urgent need to develop non-viral delivery vectors that can selectively target the bladder with minimal activation of systemic immune responses to the delivery vectors. Moreover, as genetic circuit design must be based on a patient’s genetic information, ethical concerns may arise regarding the patient’s privacy.

Notably, the genetic circuits hold considerable potential for future development. As our understanding of living organisms deepens, more genetic circuit elements will be identified. Future research in tumor therapy will gradually shift from understanding the mechanisms involved to its modification. It is anticipated that “intelligent biotherapy,” with genetic circuits as its core component, will become a new paradigm for precision tumor therapy.

## Data Availability

Not applicable.
